# Correction: Maliszewska et al. On the Photo-Eradication of Methicillin-Resistant *Staphylococcus aureus* Biofilm Using Methylene Blue. *Int. J. Mol. Sci.* 2022, *24*, 791

**DOI:** 10.3390/ijms241210221

**Published:** 2023-06-16

**Authors:** Irena Maliszewska, Anna Zdubek

**Affiliations:** Department of Organic and Medicinal Chemistry, Faculty of Chemistry, Wrocław University of Science and Technology, Wybrzeże Wyspiańskiego 27, 50-370 Wrocław, Poland; anna.zdubek@pwr.edu.pl

The authors wish to make the following corrections to this paper [1].

In the original publication, there were mistakes in Figure 4 and Figure 5 as published. The fluorescence microscopy images of biofilm formed on the glass surface by *S. aureus* 3515 were prepared by a Ph.D. student who is not a co-author of this paper. Thus, these images involve copyright issues and need to be corrected. The corrected Figure 4 and Figure 5 appear below. The authors state that the scientific conclusions are unaffected. This correction was approved by the Academic Editor. The original publication has also been updated.

## Figures and Tables

**Figure 4:**
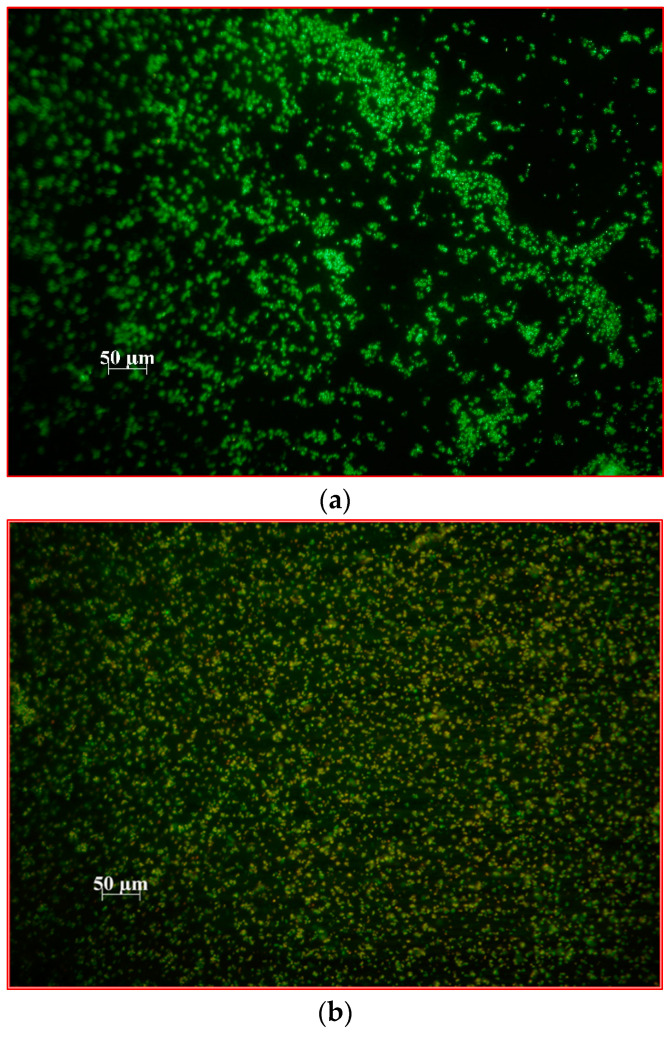

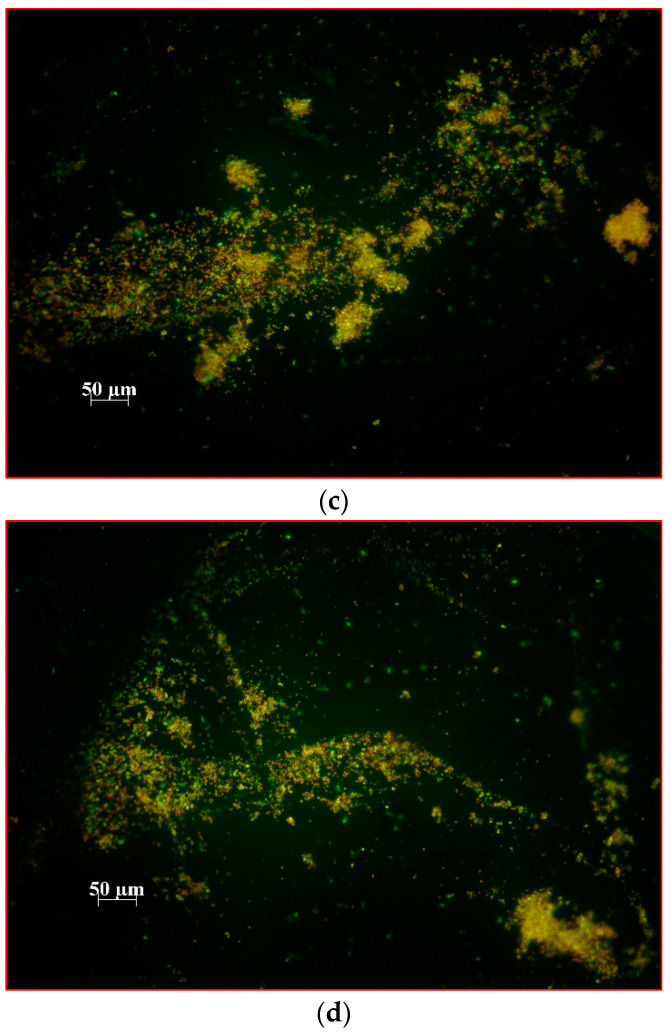
Fluorescence microscopy images of biofilm formed on the glass surface by *S. aureus* 3515 before aPDI (**a**); after photodynamic inactivation with MB alone as a photosensitizer (**b**); after photodynamic inactivation with MB+AuBNPs as a photosensitizer (**c**); after photodynamic inactivation with MB+AuChNPs as a photosensitizer (**d**). In the presence of the SYTO 9 and propidium iodide mixture, bacteria with intact cell membranes stain fluorescent green, whereas bacteria with damaged membranes stain fluorescent orange/red.

**Figure 5:**
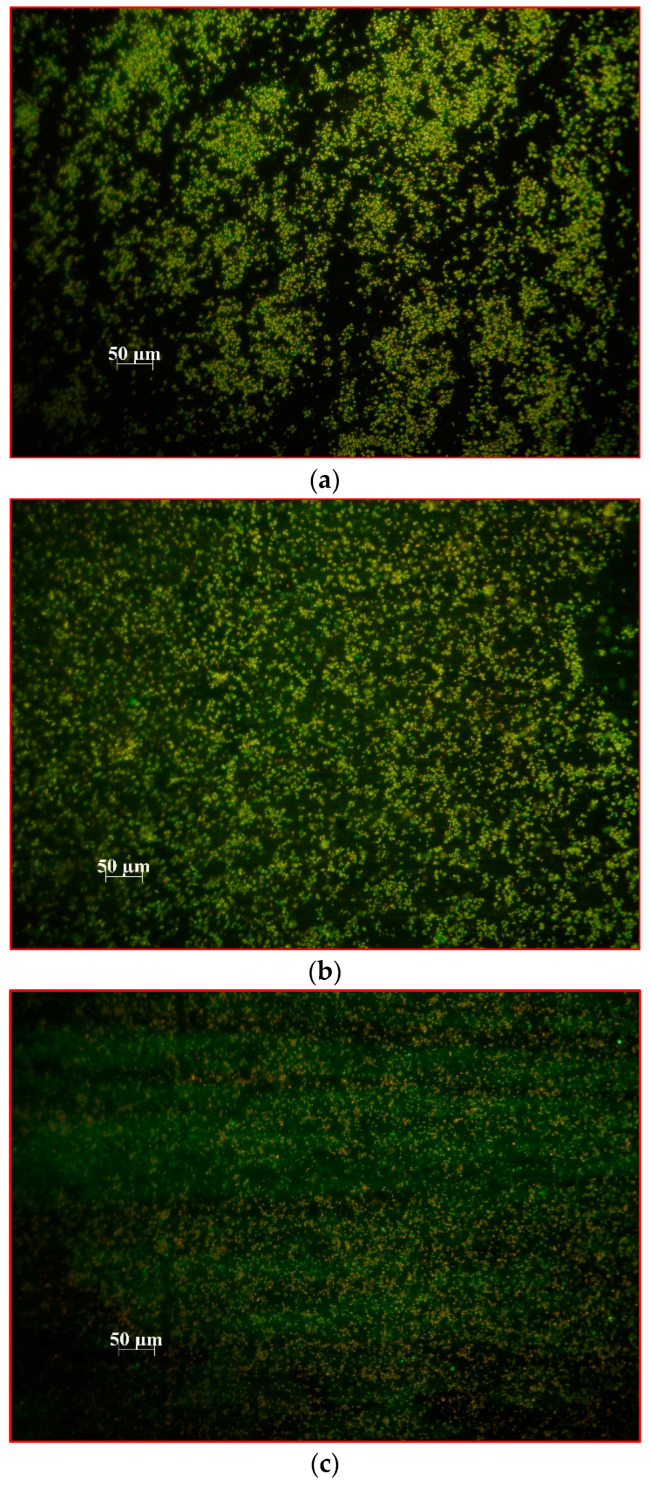
Fluorescence microscopy images of biofilm formed on the glass surface by *S. aureus* 3375 after the first (**a**), second (**b**) and third (**c**) exposures to laser light in the presence of MB alone at a nontoxic concentration of 31.25 mg L^−1^. In the presence of the SYTO 9 and propidium iodide mixture, bacteria with intact cell membranes stain fluorescent green, whereas bacteria with damaged membranes stain fluorescent orange/red.

## References

[1] Maliszewska I., Zdubek A. (2023). On the Photo-Eradication of Methicillin-Resistant *Staphylococcus aureus* Biofilm Using Methylene Blue. Int. J. Mol. Sci..

