# Supplementary material for: On the Photo-Eradication of Methicillin-Resistant Staphylococcus aureus Biofilm Using Methylene Blue
Source: Int J Mol Sci. 2023 Jan 2;24(1):791. doi: 10.3390/ijms24010791 (PMC9821080; doi:10.3390/ijms24010791)
Supplement: Supplementary file 1 [file ijms-24-00791-s001.zip › ijms-2112271-supplementary.pdf]

International Journal of
Molecular Sciences

MDPI

Supplementary Information

On the photo-eradication of methicillin-resistant *Staphylococcus aureus* biofilm using Methylene Blue

Irena Maliszewska* and Anna Zdubek

Department of Organic and Medicinal Chemistry, Faculty of Chemistry, Wrocław University of Science and Technology, Wybrzeże Wyspiańskiego 27, 50-370 Wrocław, Poland
* Correspondence: irena.helena.maliszewska@pwr.edu.pl

Susceptibility to antibiotics among the clinical isolates. Antimicrobial susceptibility to antibiotics was performed using CLSI (Clinical and Laboratory Standards Institute) broth microdilution methodology with Mueller Hinton broth (Sigma-Aldrich) [44]. The following breakpoint values were used to determine resistance (R) by microdilution method: penicillin (methicillin) R>0.125 mgL−1, gentamycin R>2 mgL^{-1}, erythromycin R>1 mgL^{-1}, tetracycline R>2 mgL^{-1}. The breakpoint is a selected concentration (mgL^{-1}) of an antibiotic that determines whether the bacteria is susceptible or resistant to the antibiotic. **MIC = minimal inhibitory concentration** was defined as the lowest concentration (mgL^{-1}) of an antibiotic that prevents the visible growth of bacteria. When the MIC is less than or equal to the susceptibility breakpoint, bacteria are considered susceptible to the antibiotic. When the MIC is greater than this value, bacteria are considered intermediate or antibiotic resistant according to the British Society for Antimicrobial Chemotherapy Resistance Surveillance Project [45].

Table S1. MICs values (mgL^{-1}) of antibiotics for *S. aureus* isolates

Isolate number	Penicillin/ methicillin	Gentamycin	Erythromycin	Tetracycline
	MIC [mgL^{-1}]*			
**3374**	6.25/0.78	0.39	0.78	1.56
**3375**	12.5/3.125	0.39	0.78	1.56
**3515**	12.5/3.125	0.39	0.78	1.56
**3690**	6.25/3.125	0.39	0.78	1.56

The dark cytotoxicity of Methylene blue and gold nanoparticles. The effect of MB and gold nanoparticles (AuBNPs and AuChNPs) on the viability of the biofilm formed by *S. aureus* isolates was studied in the dark at 37 °C. For this purpose, MB solution at a concentration of 31.25, 62.5 or 125 mgL^{-1} was dropped onto the surface of glass or steel discs with biofilms, incubated for 30 min, and then gently rinsed with deionized water. The effect of AuBNPs and AuChNPs on the viability of biofilms was studied according protocol described above except that the MB solution was replaced with colloidal gold at concentrations of 10, 20 or 40 ppm. The effect of MB and gold nanoparticles on the viability of bacterial cells was estimated using a colony-counting assay. The test glass/steel disks were washed twice with 1.5 ml of PBS to remove loose cells. Then each disc was immersed aseptically in 5 ml of PBS in a 15 ml polystyrene conical Falcon polystyrene centrifuge tube. The biofilm was removed from the disc surface by three 10-s vortex followed by 3-minute sonication using an ultrasonic homogenizer (VC505, Sonics, USA) with a power output of 50 W. The viability of the bacterial cells in the biofilm was quantified in a colony-counting assay. For this, appropriate serial dilutions were made and duplicate samples were seeded on Mueller-Hinton Agar (Sigma-Aldrich), and incubated for 24-48 hours at 37 °C. After incubation, the number of colony-forming units per cm^2 of disc surface ($CFUcm^{-2}$) was determined. The results were enumerated as log_{10} $cellscm^{-2}$ (N). To evaluate antibacterial activity, the percentage reduction (%) of bacteria was calculated as the reduction in viability $R=(N_0-N) \times 100/N_0$, where N_0 and N are the numbers of CFU at initial (1.5-4.5x10^6 $CFUcm^{-2}$) and remaining in suspension after incubation with MB or gold nanoparticles under dark conditions. Each experiment was carried out in 3 replications.

Table S2. Dark cytotoxicity of MB, AuBNPs and AuChNPs on *S. aureus* biofilms developed on the glass discs estimated using colony-counting assay

A-24-hour biofilm (biofilm formed on the surface within 24 hours)

Isolate	MB			AuBNPs			AuChNPs		
	Concentration								
	31.25 mgL^{-1}	62.5 mgL^{-1}	125 mgL^{-1}	10 ppm	20 ppm	40 ppm	10 ppm	20 ppm	40 ppm
	Viability reduction [%]								
**3374**	13.5±2.1	54.6±2.5	63.7±2.5	12.3±2.3	53.5±2.2	65.3±2.3	11.2±2.4	51.3±2.1	80.0±2.1
**3375**	12.6±2.4	36.5±2.3	71.2±2.6	12.7±2.5	16.3±2.1	21.1±2.1	11.8±2.3	38.4±2.1	64.1±2.5
**3515**	15.1±2.3	50.8±2.3	66.1±2.3	18.1±2.5	33.7±2.1	62.3±2.1	10.3±2.3	65.3±2.4	70.2±2.1
**3690**	15.3±2.5	43.4±2.1	68.3±2.3	12.3±2.5	11.3±2.6	40.3±2.0	11.4±2.8	71.2±2.3	82.5±2.2

B- 48-hour biofilm (biofilm formed on the surface within 48 hours)

Isolate	MB			AuBNPs			AuChNPs		
	Concentration								
	31.25 mgL^{-1}	62.5 mgL^{-1}	125 mgL^{-1}	10 ppm	20 ppm	40 ppm	10 ppm	20 ppm	40 ppm
	Viability reduction [%]								
**3374**	11.4±2.2	48.8±2.1	51.3±2.6	12.1±2.1	44.5±2.8	52.3±2.6	11.2±2.4	41.2±2.3	51.1±2.8
**3375**	12.1±2.3	49.1±2.3	51.3±2.5	12.2±2.4	13.0±2.4	20.7.±2.5	8.0±2.5	23.4±2.2	66.6±2.6
**3515**	13.2±2.1	36.3±2.3	71.2±2.6	14.0±2.4	36.3±2.6	54.9±2.1	10.0±2.4	41.6±2.3	44.1±2.5
**3690**	12.6±2.2	34.3±2.2	46.5±2.7	11.0±2.3	11.2±2.4	41.3±2.7	9.5±2.3	71.0±2.4	85.3±2.6

Table S3. Dark cytotoxicity of MB, AuBNPs and AuChNPs on *S. aureus* biofilms developed on the steel discs estimated using colony-counting assay

A-24-hour biofilm (biofilm formed on the surface within 24 hours)

Isolate	MB			AuBNPs			AuChNPs		
	Concentration								
	31.25 mgL^{-1}	62.5 mgL^{-1}	125 mgL^{1}	10 ppm	20 ppm	40 ppm	10 ppm	20 ppm	40 ppm
	Viability reduction [%]								
**3374**	11.1±2.4	31.8±2.7	56.4±2.3	11.4±2.2	14.3±2.6	50.0±2.5	11.4±2.1	14.1±2.5	51.2±265
**3375**	11.1±2.4	34.5±2.5	43.2±2.4	13.3±2.1	61.1±2.2	81.1±2.5	11.7±2.7	48.2±2.4	81.1±2.2
**3515**	15.1±2.5	61.3±2.5	70.2±2.6	15.1±2.3	31.1±2.4	72.2±2.5	10.2±2.6	71.1±2.4	83.2±2.4
**3690**	16.1±2.6	82.1±2.3	81.1±2.1	11.1±2.1	56.1±2.6	71.6±2.5	10.3±2.7	63.2±2.4	72.3±2.4

B-48-hour biofilm (biofilm formed on the surface within 48 hours)

Isolate	MB			AuBNPs			AuChNPs		
	Concentration								
	31.25 mgL^{-1}	62.5 mgL^{-1}	125 mgL^{-1}	10 ppm	20 ppm	40 ppm	10 ppm	20 ppm	40 ppm
	Viability reduction [%]								
**3374**	11.2±2.4	21.3±2.4	47.8±2.1	11.4±2.4	10.8±2.3	43.2±2.7	10.7±2.5	14.7±2.4	51.4±2.4
**3375**	11.3±2.6	23.4±2.4	41.6±2.2	12.1±2.4	21.6±2.4	21.3±2.7	10.8±2.7	20.5±2.5	51.1±2.5
**3515**	11.2±2.5	50.5±2.2	57.3±2.7	11.6±2.4	33.2±2.5	42.4±2.4	10.2±2.7	55.1±2.7	61.5±2.5
**3690**	12.5±2.4	63.1±2.2	71.5±2..7	11.2±2.3	51.1±2.4	70.1±274	11.2±2.2	6535±2.3	72.4±2.5

International Journal of *Molecular Sciences*

MDPI

Table S4. Photo-toxicity of MB, MB+AuBNPs and MB+AuChNPs on *S. aureus* biofilms developed on the glass discs

Isolate number	MB*		MB*+AuBNPs		MB*+AuChNPs	
	Age of biofilm [h]**					
	24	48	24	48	24	48
	Reduction in viability (log_{10} CFUcm^{-2} /[%])					
**3374**	0.91±0.03/88±1	0.55±0.02/72±2	1.15±0.03/93±4	1.09±0.03/92±3	4.32±0.005/99.995±0.001	4.41±0.05/99.96±0.01
**3375**	0.82±0.03/85±1	0.53±0.03/70±2	1.15±0.04/93.0±0.7	0.85±0.03/86±1	4.13±0.05/99.990±0.001	3.95±0.06/99.98±0.1
**3515**	1.52±0.04/97±3	0.77±0.03/83±1	2.0±0.04/99.0±0.6	1.30±0.04/95.0±0.4	3.0±0.05/99.9±0.01	1.52±0.04/97±3
**3690**	1.74±0.04/82±2	2.0±0.08/99.0±0.2	1.52±0.04/97.0±0.3	1.0±0.04/90.0±0.9	3.1±0.06/99.92±0.01	1.30±0.05/95.0±0.5
**Average reduction in the number of viable cells [%]*** **	88±2	81±1.5	99.5±1.5	90.8±1.3	99.951±0.005	97.98±0.95

Table S5. Photo-toxicity of MB, MB+AuBNPs and MB+AuChNPs on *S. aureus* biofilms developed on the steel discs

Isolate number	MB*		MB*+AuBNPs		MB*+AuChNPs	
	Age of biofilm [h]**					
	24	48	24	48	24	48
	Reduction in viability (log_{10} CFUcm^{-2} /[%])					
**3374**	0.91±0.02/88±1	0.55±0.02/72±1	2.7±0.05/99.80±0.03	0.76±0.03/83±1	3.15±0.06/99.93±0.06	1.89±0.04/98.0±0.1
**3375**	0.79±0.03/84±1	0.41±0.02/62±1	0.95±0.03/89±1	0.8±0.02/84±1	3.0±0.06/99.90±0.01	1.46±0.03/97±0.3
**3515**	1.62±0.03/98.0±0.5	0.86±0.02/86.0±0.5	2.7±0.05/99.80±0.02	1.27±0.04/95.0±0.5	3.95±0.06/99.989±0.001	1.57±0.04/97.0±0.5
**3690**	0.92±0.03/88±1	0.61±0.03/76±1	1.6±0.04/98.0±0.4	1.49±0.04/97.0±0.4	3.7±0.06/99.980±0.003	1.64±0.05/98.0±0.3
**Average reduction in the number of viable cells [%]*** **	89.5±0.8	74.0±0.8	96.65±0.36	89.8±0.7	99.948±0.021	97.5±0.3

*Concentration of MB was 62.5 mgL^{-1}; the laser light dose was 189 Jcm^{-2}

**Age of biofilm (24 h or 48 h)= biofilm formed on the surface within 24 or 48 hours

*** Average reduction in the number of viable cells [%]- this average value was calculated as the arithmetic mean of the lethality level of the strains designated as 3374, 3375, 3515, 3690

Table S6. Photo-toxicity of MB on *S. aureus* biofilms developed on the glass and steel discs

Isolate number	**Methylene Blue***			
	Glass discs		Steel discs	
	**Age of biofilm [h]****			
	**24**	**48**	**24**	**48**
	Reduction in viability ($\log_{10}$ CFUcm^{-2}/[%])			
**3374**	3.54±0.03/99.97±0.03	3.05±0.03/99.91±0.03	3.0±0.2/99.90±0.03	2.72±0.02/99.81±0.03
**3375**	4.43±0.03/99.996±0.003	4.10±0.02/99.992±0.003	4.10±0.02/99.992±0.004	3.85±0.02/99.986±0.002
**3515**	3.57±0.03/99.973±0.04	3.10±0.02/99.92±0.02	3.25±0.02/99.94±0.03	3.00±0.03/99.90±0.03
**3690**	3.75±0.04/99.98±0.02	3.25±0.03/99.94±0.02	3.30±0.02/99.95±0.03	3.00±0.01/99.90±0.02
**Average reduction in the number of viable cells [%]*****	99.980±0.020	99.940±0.024	99.946±0.023	99.899±0.020

*Concentration of MB was 125 mgL^{-1}; the laser light dose was 189 Jcm^{-2}

**Age of biofilm (24 h or 48 h)= biofilm formed on the surface within 24 or 48 hours

*** Average reduction in the number of viable cells [%]- this average value was calculated as the arithmetic mean of the lethality level of the strains designated as 3374, 3375, 3515, 3690

Table S7. Photo-eradication of *S. aureus* biofilms developed on the glass discs using MB alone, combined with removal of dead bacteria

Isolate number	Methylene Blue*	
	Age of biofilm [h]**	
	**24**	**48**
	Reduction in viability (log_{10} $CFUcm^{-2}$/[%])	
**3374**	3.75±0.04/99.98±0.03	3.54±0.03/99.97±0.03
**3375**	4.10±0.02/99.992±0.02	3.85±0.04/99.985±0.04
**3515**	3.75±0.05/99.98±0.04	3.0±0.1/99.9±0.1
**3690**	3.06±0.03/99.91±0.04	3.0±0.2/99.9±0.2
**Average reduction in the number of viable cells [%]*** **	99.965±0.030	99.94±0.09

*Concentration of MB was 31.25 mgL^{-1}; the laser light dose was 189 Jcm^{-2}
**Age of biofilm (24 h or 48 h)= biofilm formed on the surface within 24 or 48 hours
*** Average reduction in the number of viable cells [%]- this average value was calculated as the arithmetic mean of the lethality level of the strains designated as 3374, 3375, 3515, 3690

Table S8. Photo-eradication of *S. aureus* biofilms developed on the steel discs using MB alone, combined with removal of dead bacteria

Isolate number	Methylene Blue*	
	Age of biofilm [h] **	
	**24**	**48**
	Reduction in viability (log_{10} $CFUcm^{-2}$/[%])	
**3374**	3.95±0.03/99.99±0.04	3.70±0.03/99.98±0.03
**3375**	4.43±0.02/99.996±0.03	4.13±0.03/99.926±0.03
**3515**	3.50±0.05/99.969±0.004	3.01±0.03/99.91±0.02
**3690**	3.35±0.03/99.96±0.03	3.12±0.03/99.92±0.05
**Average reduction in the number of viable cells [%]*** **	99.979±0.030	99.934±0.032

*Concentration of MB was 31.25 mgL^{-1}; the laser light dose was 189 Jcm^{-2}
**Age of biofilm (24 h or 48 h)= biofilm formed on the surface within 24 or 48 hours
***Average reduction in the number of visible cell **[%]**- this average value was calculated as the arithmetic mean of the lethality level of the strains designated as 3374, 3375, 3515, 3690

Table S9. The effectiveness of removing biofilms formed by *S. aureus* MRSA isolates from glass and steel surfaces by two commercially available disinfectant products designed as ① and ②

Isolate numer	Biofilm formed on the glass discs			
	Disinfectant ①		Disinfectant ②	
	Age of biofilm [h] *			
	**24**	**48**	**24**	**48**
	Reduction in viability (log_{10} CFUcm^{-2}/[%])			
**3374**	0.74±0.02/82±2	0.45±0.03/65±3	1.72±0.02/98±2	1.20±0.03/93±3
**3375**	0.94±0.02//89±2	0.63±0.02/77±2	1.3±0.02/95±2	1.00±0.02/90±2
**3515**	0.60±0.02/75±2	0.45±0.03/65±3	0.82±0.03/85±2	0.70±0.03/80±3
**3690**	0.62±0.03/76±3	0.52±0.03/70±3	1.09±0.02/92±2	0.82±0.03/85±3
**Average reduction in the number of viable cells [%]****	80±2	69±3	92±2	87±3
Isolate numer	Biofilm formed on the steel discs			
	Disinfectant ①		Disinfectant ②	
	Age of biofilm [h] *			
	**24**	**48**	**24**	**48**
	Reduction in viability (log_{10} CFUcm^{-2}/[%])			
**3374**	0.15±0.04/30±4	0.09±0.05/26±5	0.38±0.03/59±4	0.28±0.03/48±3
**3375**	0.37±0.03/57±3	0.26±0.04/45±4	0.79±0.03/84±3	0.60±0.03/75±3
**3515**	0.14±0.04/29±4	0.09±0.04/20±4	0.28±0.03/48±3	0.26±0.04/45±4
**3690**	0.49±0.03/68±3	0.39±0.03/60±3	0.77±0.02/83±2	0.70±0.02/80±2
**Average reduction in the number of viable cells [%]****	46±3	38±4	68±3	62±3

Disinfectant ①=100 g of this product contained 25 g of ethanol (94%), and 35 g of propan-1-ol
Disinfectant ②= this product contained quaternary ammonium salts (alkyl (C12-14 benzyldimethylammonium chloride
*Age of biofilm (24 h or 48 h) **-**biofilm formed on the surface within 24 or 48 hours
Average reduction in the number of visible cell **[%]- this average value was calculated as the arithmetic mean of the lethality level of the strains designated as 3374, 3375, 3515, 3690
